# Ginkgolic acid and anacardic acid are reversible inhibitors of SARS-CoV-2 3-chymotrypsin-like protease

**DOI:** 10.1186/s13578-022-00806-6

**Published:** 2022-05-19

**Authors:** Dongsheng Li, Gangan Yan, Wenwen Zhou, Shuyi Si, Xiaoping Liu, Jing Zhang, Yan Li, Yunyu Chen

**Affiliations:** 1 Institute of Medicinal Biotechnology, Chinese Academy of Medical Sciences and Peking Union Medical College, Beijing, 100050 China; 2 Institute for Drug Screening and Evaluation, Wannan Medical College, Wuhu, 241002 China

**Keywords:** SARS-CoV-2, 3CL protease inhibitor, Non-covalent, Reversible, Mixed-inhibition manner, Ginkgolic acid, Anacardic acid

## Abstract

Because of the emerging variants of severe acute respiratory syndrome coronavirus 2 (SARS-CoV-2) in different regions of the world, the battle with infectious coronavirus disease 2019 (COVID-19) caused by SARS-CoV-2 has been seesawing. Therefore, the identification of antiviral drugs is of particular importance. In order to rapidly identify inhibitors for SARS-CoV-2 3-chymotrypsin-like protease (3CL^pro^), an enzyme essential for viral replication, we combined the fluorescence polarization (FP) technique with biotin-avidin system (BAS) and developed a novel sandwich-like FP screening assay. Through high-throughput screening, two hits of 3CL^pro^ inhibitors, ginkgolic acid (GA) and anacardic acid (AA) were identified, which showed IC_50_ values of 11.29 ± 0.48 and 12.19 ± 0.50 μM, respectively. Their binding modes were evaluated by HPLC-Q-TOF–MS. There was no mass increase detected for SARS-CoV-2 3CL^pro^ incubated with either GA or AA, indicating the absence of covalent adducts. The kinetic analysis clearly demonstrated that both GA and AA inhibit SARS-CoV-2 3CL^pro^ via reversible and mixed-inhibition manner. Our results argue against conclusion that GA and AA act as irreversible and covalent inhibitors against SARS-CoV-2 3CL^pro^, which is based on the studies by Chen et al*.*

## Dear Editor,

Although much progress has been made in the surveillance and control of coronavirus disease 2019 (COVID-19) pandemic around the world since its outbreak in 2019, the pathogen severe acute respiratory syndrome coronavirus 2 (SARS-CoV-2) has been undergoing mutations continuously, raising the concerns that the SARS-CoV-2 variants may gain resistance to the current SARS-CoV-2 neutralizing antibodies, vaccines, RNA-dependent RNA polymerase (RdRp) inhibitors and 3-chymotrypsin-like protease or main protease (3CL^pro^ or M^pro^) inhibitors [1–4]. If nothing else, SARS-CoV-2 has taught us that widespread proliferation, low fidelity genome synthesis, and selective pressure will quickly produce drug resistant phenotypes [3]. Therefore, there is still an urgent need to develop safe, effective, and affordable prevention/treatment agents for SARS-CoV-2 infection and future drug-resistance.

**Fig. 1:**
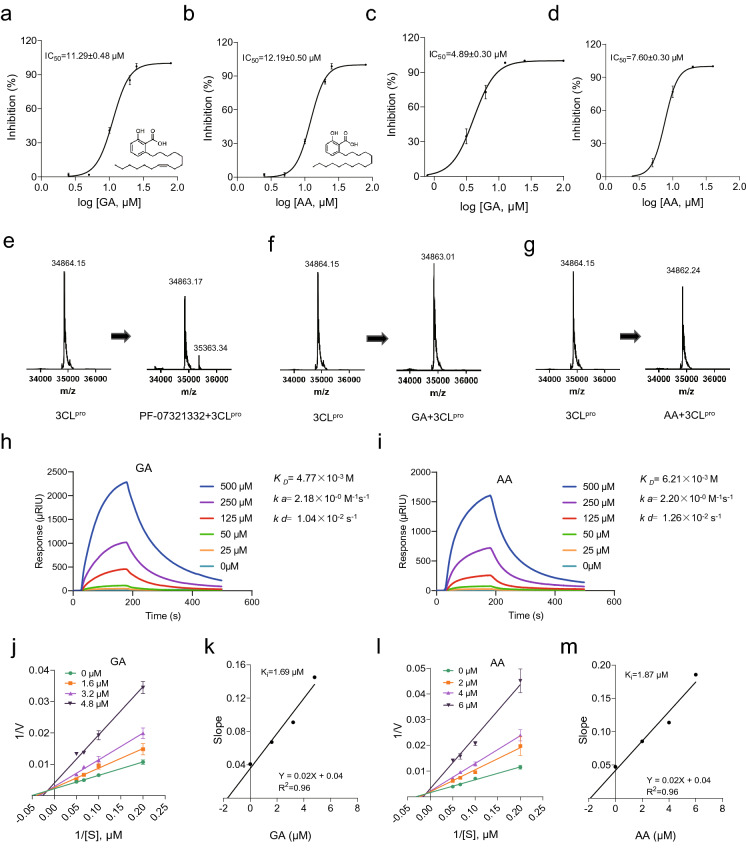
The inhibitory activity and mechanism of ginkgolic acid (GA) and anacardic acid (AA) against SARS-CoV-2 3CL^pro^. **a**, **b** Dose-dependent inhibition of SARS-CoV-2 3CL^pro^ by GA (**a**) and AA (**b**) using sandwich-like fluorescence polarization (FP) assay. As described previously [5], the mixture of SARS-CoV-2 3CL^pro^ (0.4 μM) and GA or AA with concentrations ranging from 2.5 to 80 μM was preincubated for 35 min at room temperature (RT), then 40 nM FP tracer (FITC-AVLQSGFRKK-Biotin) was added into the mixture to initiate proteolytic reaction. After addition of avidin, the millipolarization unit (mP) value was measured to calculate the IC_50_ using GraphPad Prism 8.0. Three independent experiments were performed. **c**, **d** IC_50_ plots from in vitro fluorescence resonance energy transfer (FRET)-based enzymatic assay against SARS-CoV-2 3CL^pro^ of GA (**c**) and AA (**d**). SARS-CoV-2 3CL^pro^ was incubated in the reaction buffer with various concentrations of GA or AA at RT for 30 min. Then the enzymatic reaction was initiated by adding MCA-AVLQSGFRLys(Dnp)-Lys-NH2 as the fluorescently labeled substrate. After the RFU value monitored by a microplate reader (BioTek), the efficacy of two protease inhibitors was evaluated in GraphPad Prism 8.0. The results are average ± SD of three repeats. **e–g** Binding mode analysis between PF-07321332 (**e**), GA (**f**) or AA (**g**) and SARS-CoV-2 3CL^pro^ using HPLC-Q-TOF MS. According to the published protocol [10], purified SARS-CoV-2 3CL^pro^ (5 μM) was incubated with or without PF-07321332, GA or AA (500 μM) in TBS (10 mM Tris, 50 mM NaCl pH 8.0) at RT for 30 min. The desalted samples were analyzed by the quadrupole time-of-flight (Q-TOF) mass spectrum (Agilent, USA) for detecting the molecular weight of intact 3CL^pro^. Mass spectrum were deconvoluted using Mass Hunter software (Agilent), and maximum entropy was performed for deconvolute algorithm. **h**, **i** Evaluation of binding activity of GA (h) and AA (i) to SARS-CoV-2 3CL^pro^ using SPR. The affinity of GA and AA (25, 50, 125, 250, and 500 μM) to SARS-CoV-2 3CL^pro^ was examined separately by real-time SPR spectroscopy, with 20 μL SARS-CoV-2 3CL^pro^ (1.6 mg/mL) in 10 mM NaAc buffer (pH5.5) immobilized on the flow cell of the sensor chip CM5. The kinetics parameters (*k*_*a*_, *k*_*d*_ and *K*_*D*_) were calculated using the analyte binding kinetic curve. **j**, **l** The Lineweaver–Burk plots for analysis the inhibition mechanisms of GA (**j**) and AA (**l**) against 3CL^pro^ using the FRET assay. **k**, **m** The secondary plots for the inhibitory constant (*Ki*) values of GA (**k**) and AA (**m**) in the FRET substrate

Several viral proteins, including 3CL^pro^, Papain-like protease (PL^pro^) and RdRp have been prioritized as promising anti-COVID-19 drug targets. Among the three viral proteases, 3CL^pro^ appears to be a high-profile drug target for the development of broad-spectrum antivirals: first, 3CL^pro^ plays an essential role in coronavirus replication by cleaving the viral polyproteins at more than 11 sites; second, 3CL^pro^s have relatively high sequence similarity within each CoV group; moreover, 3CL^pro^ has an unique substrate preference for glutamine at the P1 site (Leu-Gln↓(Ser,Ala,Gly)), a feature that is absent in closely related host proteases; last but not least, up to now, none of the 25 most common 3CL^pro^ mutants involve residues in the active site or at the dimerization interface [3]. Even with several exceptions, including P132H, the resulting amino acid is often similar in size and physicochemical properties, such as K → R, which does not compromise small-molecule drug inhibition [3]. Therefore, it is feasible to design 3CL^pro^ inhibitors with high selectivity and high potential to monitor drug-resistance. Our primary goal was to identify lead compounds targeting SARS-CoV-2 3CL^pro^ using our newly established sandwich-like FP screening assay [5]. Among 3000 tested compounds, ginkgolic acid (GA) and anacardic acid (AA) displayed the most potent inhibitory effects on the hydrolytic activity of SARS-CoV-2 3CL^pro^. As illustrated in Fig. 1a, SARS-CoV-2 3CL^pro^ was inhibited by GA in a dose-dependent manner, with half maximal inhibitory concentration (IC_50_) value of 11.29 ± 0.48 μM. The IC_50_ value of AA against SARS-CoV-2 3CL^pro^ was 12.19 ± 0.50 μM (Fig. 1b), showing similar inhibitory efficiency as GA. Subsequently, we confirmed the proteolytic inhibition of GA and AA against 3CL^pro^ using fluorescence resonance energy transfer (FRET) assay. The IC_50_ values of GA and AA towards 3CL^pro^ were 4.89 ± 0.30 μM (Fig. 1c) and 7.60 ± 0.30 μM (Fig. 1d), respectively, which were similar to the previously published results [6, 7].

Covalent inhibitors usually use electrophilic moieties, prominently nitrile, disulfide or cyanoacrylate to react with a corresponding site in its target [8]. The majority of current reported SARS-CoV-2 3CL^pro^ inhibitors are peptidomimetic covalent inhibitors with a reactive warhead such as ketone, aldehyde or ketoamide [9]. A highlighting milestone is Nirmatrelvir (PF-07321332), a covalent inhibitor carrying a nitrile warhead that targets SARS-CoV-2 3CL^pro^, which plus Ritonavir has received its first conditional authorization on 31 December 2021 for the treatment of COVID-19 in the United Kingdom [10]. Nirmatrelvir has also been authorized for emergency use in the USA (December 2021), and more recently received a conditional authorization in the EU (January 2022). Inspired by this fact, we used PF-07321332 as a positive control to demonstrate that the covalent conjugate can be detected by our developed mass spectrum assay. Notably, the presence of a mass shift of 500 Da after treatment with PF-07321332 indicated a covalent adduct formation (Fig. 1e), in agreement with the results presented in the previous study [10]. Considering the lack of reactive group in GA and AA, covalent inhibition against 3CL^pro^ is usually unlikely. To test this idea, we compared the molecular weights of 3CL^pro^ before and after its incubation with GA or AA using HPLC-Q-TOF–MS. A MS peak with a mass value of 34,863.01 (Fig. 1f)/34,862.24 Da (Fig. 1g) was detected for SARS-CoV-2 3CL^pro^ incubated with GA or AA, respectively. These numbers were equal to the molecular weight of SARS-CoV-2 3CL^pro^ alone (34,864.15 Da) (Fig. 1f, g), indicating non-covalent conjugation with either GA or AA. In conclusion, our results suggested that neither GA nor AA covalently reacted with SARS-CoV-2 3CL^pro^.

To further understand the nature of non-covalent interaction between SARS-CoV-2 3CL^pro^ and GA or AA at the molecular level, surface plasmon resonance (SPR)-based binding assay was used to measure the affinity constant and characterize these interactions. Although GA could increase the real refractive index unit (RIU) response of SARS-CoV-2 3CL^pro^ in a dose-dependent manner, the *K*_*D*_ was 4.77 × 10^–3^ M (Fig. 1h), indicating poor affinity and reversible binding mode between GA and SARS-CoV-2 3CL^pro^. Very similar results were obtained regarding the binding of AA to SARS-CoV-2 3CL^pro^ with *K*_*D*_ of 6.21 × 10^–3^ M (Fig. 1i). Meanwhile, both GA and AA exhibited fast association rate (*ka*) and fast dissociation rate (*kd*) values in binding kinetics curves (Fig. 1h, i).

It is well documented that reversible inhibitors bind to enzymes by non-covalent bonds. The non-covalent adduct formation between SARS-CoV-2 3CL^pro^ and GA or AA indicated that GA and AA are likely to exert inhibitory effects via reversible inhibition. Therefore, kinetic analysis was performed to investigate the inhibitory mechanisms of GA and AA using FRET assay. Based on enzyme kinetics analysis, the slopes and intercepts of the reciprocal Lineweaver–Burk plots elevated with the increase in inhibitor concentration, which is inconsistent with the three main types of inhibition, competitive, noncompetitive, or uncompetitive (Fig. 1j, l). The intersection of each trend line in the second quadrant suggested a mixed-type inhibitory mode, implying that these agents may bind this target enzyme at both catalytic active site and non-catalytic site. The inhibitory constant (*Ki*) values of GA and AA were 1.69 and 1.87 μM, respectively (Fig. 1k, m). Overall, these results suggested that GA and AA act as reversible and mixed-type inhibitors against SARS-CoV-2 3CL^pro^, which is supported by the previous finding [6].

In summary, our data provide insights into the binding modes between SARS-CoV-2 3CL^pro^ and GA or AA, which is non-covalent, reversible and mixed-type of inhibition. Our results are inconsistent with a previously report about the covalent binding between SARS-CoV-2 3CL^pro^ and GA or AA [7]. Regardless, these compounds are promising candidates worthy of structure modification for the treatment of COVID-19.

## Data Availability

All data generated or analyzed during this study are included in this article.

## Abbreviations

GA: Ginkgolic acid
AA: Anacardic acid
BAS: Biotin-avidin system
COVID-19: Coronavirus disease 2019
FITC: Fluorescein isothiocyanate
FP: Fluorescence polarization
FRET: Fluorescence resonance energy transfer
MCA: 7-Methoxycoumarin-4-acetic acid
mP: Millipolarization unit
3CL^pro^: 3-chymotrypsin-like protease
RIU: Refractive index unit
RFU: Relative fluorescence units
SARS-CoV-2: Severe acute respiratory syndrome coronavirus-2
SPR: Surface plasmon resonance
